# Using the Pan American Health Organization Digital Conversational Agent to Educate the Public on Alcohol Use and Health: Preliminary Analysis

**DOI:** 10.2196/43165

**Published:** 2023-04-06

**Authors:** Maristela Goldnadel Monteiro, Daniela Pantani, Ilana Pinsky, Thiago Augusto Hernandes Rocha

**Affiliations:** 1 Department of Noncommunicable Diseases and Mental Health Pan American Health Organization Washington, DC United States; 2 Department of Health Analysis, Metrics and Evidence Pan American Health Organization Washington, DC United States

**Keywords:** alcohol use, alcohol risk assessment, digital health worker, artificial intelligence, health literacy, digital health, chatbot, misinformation, online health information, digital health education, alcohol use, health risk, COVID-19

## Abstract

**Background:**

There is widespread misinformation about the effects of alcohol consumption on health, which was amplified during the COVID-19 pandemic through social media and internet channels. Chatbots and conversational agents became an important piece of the World Health Organization (WHO) response during the COVID-19 pandemic to quickly disseminate evidence-based information related to COVID-19 and tobacco to the public. The Pan American Health Organization (PAHO) seized the opportunity to develop a conversational agent to talk about alcohol-related topics and therefore complement traditional forms of health education that have been promoted in the past.

**Objective:**

This study aimed to develop and deploy a digital conversational agent to interact with an unlimited number of users anonymously, 24 hours a day, about alcohol topics, including ways to reduce risks from drinking, that is accessible in several languages, at no cost, and through various devices.

**Methods:**

The content development was based on the latest scientific evidence on the impacts of alcohol on health, social norms about drinking, and data from the WHO and PAHO. The agent itself was developed through a nonexclusive license agreement with a private company (Soul Machines) and included Google Digital Flow ES as the natural language processing software and Amazon Web Services for cloud services. Another company was contracted to program all the conversations, following the technical advice of PAHO staff.

**Results:**

The conversational agent was named Pahola, and it was deployed on November 19, 2021, through the PAHO website after a launch event with high publicity. No identifiable data were used and all interactions were anonymous, and therefore, this was not considered research with human subjects. Pahola speaks in English, Spanish, and Portuguese and interacts anonymously with a potentially infinite number of users through various digital devices. Users were required to accept the terms and conditions to enable access to their camera and microphone to interact with Pahola. Pahola attracted good attention from the media and reached 1.6 million people, leading to 236,000 clicks on its landing page, mostly through mobile devices. Only 1532 users had a conversation after clicking to talk to Pahola. The average time users spent talking to Pahola was 5 minutes. Major dropouts were observed in different steps of the conversation flow. Some questions asked by users were not anticipated during programming and could not be answered.

**Conclusions:**

Our findings showed several limitations to using a conversational agent for alcohol education to the general public. Improvements are needed to expand the content to make it more meaningful and engaging to the public. The potential of chatbots to educate the public on alcohol-related topics seems enormous but requires a long-term investment of resources and research to be useful and reach many more people.

## Introduction

Alcohol consumption is a major global risk factor for disability and death, responsible for an estimated 3 million deaths (5.3% of all deaths) in 2016 [1]. This includes noncommunicable and infectious diseases, injuries, and secondary harms to others rather than the drinkers themselves. Broadly, alcohol consumption contributes to wider societal harms connected to crimes, road injuries, the loss of productivity, and alcohol-related violence [2,3]. Although these harms are widespread, socioeconomically disadvantaged populations are especially impacted [4,5], making alcohol a central driver of inequalities. The recognition that alcohol, beyond health, brings significant social, economic, and environmental losses to individuals and society at large renders it a cross-cutting obstacle to achieving many areas of the United Nations 2030 Sustainable Development Agenda [6].

At the same time, the production and sale of alcoholic beverages represent a highly lucrative economic activity, led, in a significant proportion, by transnational corporations and powerful trade groups. In 2017, the sale of alcohol reached more than US $1.5 trillion globally, with major investments on marketing and public relations strategies [7]. In this way, alcohol policy became an area contested in complex ways [8]. Misconceptions about alcohol products and alcohol use have been an issue with many different roots [9], including the dissemination of specific framings about alcohol harms, such as misrepresentations, as one of the strategies of the alcohol industry to shape the public opinion, bringing a vision consistent and supportive of their underlying commercial interests [10]. In broad terms, the economic sector framing tends to interpret alcohol use and harm as a matter of personal responsibility and to promote measures that are likely to have little or no impact [11]. The impact of alcohol on cancer, for instance, is an example in which the literature has already shown that alcohol is a carcinogenic substance, whereas the alcohol industry questions and distorts this information to the public [12-14]. Reports from the industry concerning the risks associated with alcohol consumption tend to present a mixed combination of accurate and inaccurate data to create an uncertain scenario and doubt [15-19]. This strategy is known as strategic ambiguity, in which material from social aspects or public relations organizations significantly misrepresents the evidence to minimize the risks associated with alcohol consumption [15-17]. Petticrew et al [9] highlighted that “it is well-documented that alcohol, tobacco, and other harmful commodities industries focus on the multifactorial etiology of many health conditions, to distract from the independent harmful effects of those commodities.”

The public health evidence, on the other hand, points clearly to solutions to alcohol harms that focus on population-level regulations such as taxation, decreased availability of alcohol, and the restriction of marketing. Health education and mass media campaigns per se have not been shown to be effective in reducing alcohol problems at the population level, particularly when they compete with the massive marketing of alcohol [20].

The use of digital solutions to improve alcohol health literacy and scale up support for individuals to reduce their alcohol consumption has been discussed for many years [21-23]. Among different emerging technologies, such as chatbots, apps, geotagging, and many others, digital health agents have the potential to provide reliable and timely information quickly and assess and support behavioral change to reduce the harmful use of alcohol to millions of people [21].

Although the framing of alcohol information by the alcohol industry and misconceptions about alcohol use are an ongoing and concerning issue to public health [12,17,20], during the COVID-19 pandemic, additional and more blatant misinformation, not necessarily connected to apparent economic interests, began to become widespread [18,19,24]. Claims that drinking alcohol could prevent COVID-19 infection were pervasive in several countries [25]. A population-based phone survey of adults in Hong Kong found that this kind of health misinformation spread through social media was associated with self-reported increases in alcohol consumption [24]. In Iran, a country where the consumption of alcohol is prohibited, the false belief that it would cure or prevent the infection resulted in more than 700 deaths due to toxic methanol (present in adulterated alcohol products sold illegally) ingestion [26]. False rumors that the frequent consumption of concentrated ethanol could kill the coronavirus were prevalent enough for several credible public health institutions, including the World Health Organization (WHO) and the Pan American Health Organization (PAHO), to put out statements trying to dispel this myth [27].

The pandemic led to an exponential increase in the use of social media for socialization, as well as by the alcohol industry to promote alcohol sales and use. Web-based alcohol sales and home deliveries are reported to have increased upon the closures of public places where alcohol was typically sold. As a result, the existing low levels of health literacy [28] on alcohol and health, combined with the increased use of social media platforms such as Facebook, Twitter, Instagram, YouTube, WhatsApp, and podcasts [29], led to an unattainable situation requiring new and fast ways of communicating with the public using reliable and trusted sources of information.

Chatbots and conversational agents are a result of programmed algorithms that interact with users using natural language, either by text or voice, presenting capabilities to support natural language conversational interactions [30,31]. A chatbot can offer a wide range of functionality that can be easily customized to address different topics, including the risk assessment of patients and employees, information dissemination by answering common questions, surveillance, and integration within an existing infrastructure [30]. Chatbots and conversational agents therefore became an important piece of the WHO response to the COVID-19 pandemic. They were developed to quickly disseminate evidence-based information and to counter misinformation about COVID-19; the effectiveness of vaccines; and other health topics, including tobacco and mental health. Chatbots and digital conversational agents have been seen as a way to broaden the WHO’s reach and to facilitate the communication with a variety of users globally [32-34].

In 2021, following the launch of Florence (a chatbot [35]) by the WHO, PAHO decided to create its first conversational agent, named Pahola, to interact with anyone interested in learning about the impact of alcohol use on health and the alcohol-related risks to one’s self and others; assessing one’s own alcohol consumption risk; and supporting those interested in reducing their drinking. The main goal of Pahola was to democratize the delivery of scientifically validated alcohol health information and health promotion and contribute to the achievement of 1 billion more healthy people, as proposed by the WHO Thirteenth General Programme of Work [36]. This paper aims to describe the development of Pahola to combat misinformation and promote alcohol health literacy.

## Methods

### Overview

From March 2021 until November 2021, PAHO’s alcohol technical team, a group of external alcohol experts, and a creative marketing agency worked to create Pahola. Around 30 people, including PAHO staff and external alcohol experts, were consulted to decide on the facial characteristics, gender, tone of voice, apparent age, clothing, and personality traits. The decision on the name Pahola was made to relate to PAHO’s acronym and make it unique.

PAHO secured a nonexclusive worldwide license from a technology company (Soul Machines) to use their Human OS ecosystem, which enables human-like interactions between a digital person and users *via* an application. The agreement allowed PAHO to use up-to-date technology to enhance the interaction between people and the agent. They include the ability of the digital agent to express specific emotions triggered through words and phrases and the presence of algorithms to process natural language and correctly interpret users’ requests. Seven technology modules were needed to develop and deploy Pahola:

1. Facial sentiment analysis of end-user video stream
2. Autonomous animation of a digital person with cognitive models
3. 3D rendering cloud architecture
4. WebRTC video and audio streaming front end
5. Natural language processing (NLP)
6. Speech-to-text conversion
7. Text-to-speech synthesis module

Each module described above performed a group of tasks to automatically process the audio or text inputs provided by the end users. The core engine responsible for processing the natural language inputs received by Pahola was integrated using Google Dialog Flow ES for NLP. Thus, modules 5, 6, and 7 are responsible for understanding the user’s intention or inquiry by analyzing their utterance and selecting the proper prewritten response from a finite set of responses. Once a match between processed audio input provided by the end user and the dialog corpus linked to the conversational agent is found, Pahola selects predefined information to be shared. For integrating the NLP core processing engine with the predefined set of questions, matching phrases were provided as part of building the conversation (for example, variations of phrases provided by PAHO on alcohol-related topics and variations of responses to the same questions).

Over 100 different alcohol topics (the same for all languages) were incorporated into Pahola to build the conversation flow, generating approximately 1500 phrases to match patterns between the NLP core engine and the alcohol-related corpus (ie, the conversation flow content). To build the conversations that were incorporated into Pahola’s corpus, PAHO staff provided the content topics and evidence-based information materials to the programmers and reviewed all content for accuracy. The main sources of information used were WHO reports, recent scientific literature reviews, and informal consultations with international experts. The conversation design started with a welcome message and a rapport followed by three main branches that users could choose to start the interaction:

1. Alcohol and health topics (“alcohol effects branch”), which comprised general information about the impact of alcohol on people’s health and alcohol health literacy;
2. Assessing the user’s own risk from drinking by completing a validated international questionnaire (Alcohol Use Disorder Identification Test; “quiz branch”); receiving feedback on the results; and if willing, receiving guidance on reducing their risk; and
3. Seeking treatment resources to quit or reduce drinking (“quit branch”).

Each branch was then divided into nodes where similar questions were grouped by themes (for instance, “alcohol and pregnancy”). The alcohol effects branch was composed of 49 nodes, the quit branch was composed of 18 nodes, and the quiz branch was composed of 22 nodes.

In any branch, users could ask different questions that could lead them to a different branch or a different node (except for the quiz and quit branches, which had a more defined path in terms of nodes that requested users to access those branches from the start to end). In addition, phrases considered “small talk” were used to enhance empathy and improve user engagement with the digital agent. The training also included variations of phrases and responses that were part of the conversation built. An “elegant error” feature was incorporated in Pahola’s reactions to handle situations in which a match between the audio or text input and the corpus branch could not be obtained. The conversation development, translation, training, and retraining took around 4 months. PAHO revised and approved all content.

The acceptance of the legal terms and conditions was required before the user could speak to Pahola, to allow the use of the microphone and camera for the conversations between the digital agent and the user.

Additionally, PAHO developed 13 fact sheets in 2021 in English, Spanish, and Portuguese on the main alcohol topics related to the information Pahola provided, which are available through PAHO’s website. Finally, a communication strategy was developed to increase Pahola’s visibility and to complement an alcohol awareness campaign focused on alcohol-related harms, launched 2 weeks before Pahola, with the slogan “Live Better, Drink Less.”

To assess the capabilities of Pahola, we used access metrics using Google Analytics and the number of interactions as outcome metrics. Data on users’ interactions were anonymously collected through Amazon Web Services and Dialog Flow (identifiable data are not retained or accessible to anyone) and accessed through the native Pahola dashboard (from the private company that has the proprietary software).

### Ethical Considerations

The study did not require ethical review as it did not involve identifiable data from users.

## Results

Pahola was deployed on November 19, 2021, during a web-based event organized by PAHO, in which high-level management and technical staff from PAHO and the WHO, experts from the alcohol field, and over 1000 registered participants participated. It is currently available through PAHO’s web page [37], and it can be accessed from any device at no cost to users.

Pahola was featured in many news portals and websites throughout Latin America and the Caribbean. For 2 months (from November 19, 2021, to January 16, 2022), the communication strategy to promote Pahola generated 1500 mentions on digital channels; 12,000 engagements on PAHO’s social media, with 1.6 million people being directly reached by its content; and 61 million impressions. The channels used included Twitter, Facebook, Instagram, and Google’s extended network through DV360 ads.

**Figure 1:**
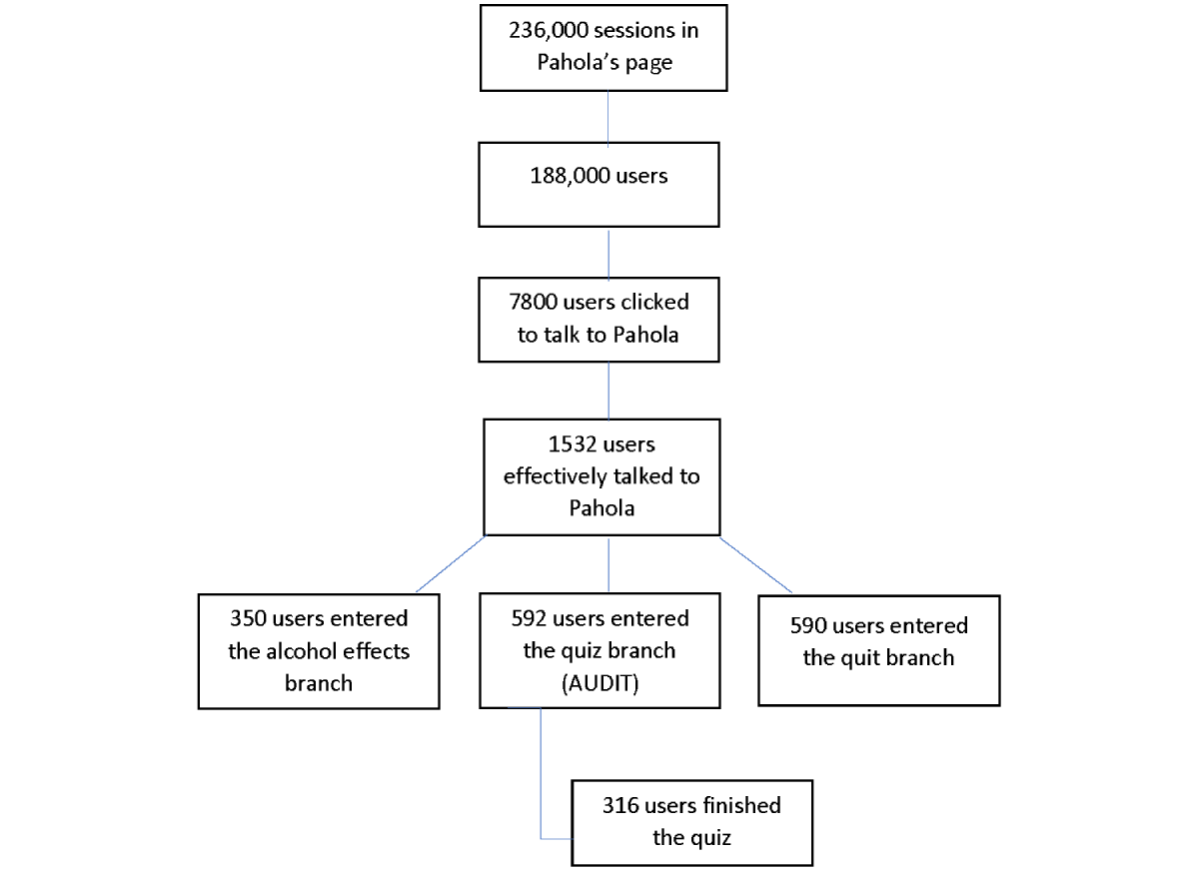
How users accessed Pahola's content (from November 19, 2021, to January 16, 2022).

The preliminary results from the first 2 months (from November 19, 2021, to January 6, 2022) after its launch showed approximately 236,000 views on Pahola’s landing page (on PAHO’s website) and 188,000 users (unique visits; see Figure 1). Pahola’s page was available in 3 languages: Spanish was the most accessed page (120,360/236,000, 51%), followed by English (68,440/236,000, 29%) and Portuguese (47,200/236,000, 20%). Around 1532 (0.8% of the 188,000 unique visitors) users effectively engaged in conversation with Pahola, mostly in Spanish (90,240/188,000, 48%), followed by English (69,560/188,000, 37%) and Portuguese (28,200/188,000, 15%).

Most people (200,600/236,000, 85%) accessed Pahola’s landing page through their mobile phone, 13% (37,440/236,000) accessed from desktop computers, and 2% (5760/236,000) accessed from tablets. This could partially explain the major drop-off observed from the number of users who landed on the page to the number of users who effectively talked to Pahola, as after the launch, an issue with mobile access from PAHO’s page was detected (on mobile devices, once one clicked to talk to Pahola, they were sent to a web page that was not showing Pahola prominently and could have led to most users giving up on interacting with it). This was corrected but did not immediately increase the number of effective interactions.

The top 10 countries that accessed the page in all languages were, in order, Argentina, the United States, Colombia, Brazil, Uruguay, Canada, Haiti, Dominican Republic, Costa Rica, and Mexico. The preferred internet browser language of users who accessed Pahola’s page was Spanish (132,160/236,000, 56%), followed by English (66,080/236,000, 28%), Portuguese (21,240/236,000, 9%), and French (14,160/236,000, 6%). The age and gender information were captured by the Google Analytics features for 25% (47,000/188,000) of the users only (Pahola asked about age ranges and not directly about the individual’s age). Regarding their sex, approximately 62% (29,140/47,000) of users were female, and 38% (17,860/47,000) were male. The main age range of users was 35-44 years (12,690/47,000, 27%), 25-34 years (7520/47,000, 16%), and 45-54 years (7520/47,000, 16%).

The average time a user spent talking to the digital agent was 5 minutes 16 seconds, ranging from 5 minutes 45 seconds in Spanish to 4 minutes 35 seconds in English. Overall, the most accessed branches in all languages at the start was the quiz branch (608/1532, 39.7%), followed by the quit branch (564/1532, 36.8%) and the alcohol effects branch (360/1532, 23.5%).

Users in all languages were the most interested in the node “What is the nutritional value of alcohol?” followed by “How healthy is it to drink?” and “Alcohol, the brain, and behavior.” There were, however, variations among languages. Spanish and English users were also very interested in the “Alcohol, sex and pregnancy” and “Social drinking” nodes. The “Is alcohol good for me?” and “Alcohol and age” nodes had good access from Portuguese users.

## Discussion

### Principal Findings

Pahola was the first digital conversational agent dedicated to alcohol-related topics. Pahola provided customized and scientifically validated information to address alcohol harms to a little over 1500 users, which was somewhat disappointing given its unlimited reach. It is also surprising to see that the branch with the most information on alcohol and health (alcohol effects branch) was the least preferred by users, who were more interested in knowing their risk or quitting or changing their drinking habits from the start. At the same time, the average time spent interacting with Pahola was considered high, compared with the usual 15-30 seconds used as a measure of success when interacting with a user. This may be an indication of its success as a conversational agent, but more research is needed to assess this. It remains unclear why, out of 236,000 people landing on its page, only 1% interacted with Pahola. Issues such as accessibility, the fear of the new technology, the requirement to access the camera and microphone to have an interaction, difficulties with language understanding, and connectivity, among others, could all have played a role.

The usage metrics obtained by Pahola can be considered a proxy of the acceptance of a smart conversation agent to perform tasks dedicated to promoting alcohol-related health literacy. The development of pioneer solutions such as Pahola is marked by the need to accommodate new evidence and adjust the parameters linked to the chatbot. The use of chatbots as tools to address health challenges is still incipient. As of September 2022, only 165 works in PubMed mentioned the findings of research initiatives based on chatbots.

Although Pahola could serve as a health educator on topics related to alcohol, not only in the Americas but globally, it is too early to say that it was successful given the relatively low levels of actual interaction. The development of health education and traditional mass campaigns to increase the awareness of the negative effects of alcohol is ineffective in counteracting the massive and pervasive alcohol marketing, which has messages that contradict those of health educators. Thus, the use of chatbots can be considered an opportunity to tackle the pervasive consequences of alcohol use misinformation [15,24]. The use of smart conversational agents enables longer and customized conversations related to alcohol anytime people want and need, anonymously and confidentially [38]. Artificial intelligence and cloud solutions allow the development of scalable chatbots with custom features to handle different needs in terms of demand.

Smart agents such as Pahola can potentially enhance other efforts to educate the public on general or specialized issues related to alcohol, such as understanding what a standard drink is, understanding how much alcohol increases a person’s risk of consequences when seeking treatment, and other services. Recommendations regarding clinical guidelines and alcohol and cancer risk can also be addressed at a much lower cost than regular interventions and with greater reach.

Although we have not yet assessed what people learned from the interactions with Pahola, all content was evidence-based. Future studies can compare its performance to the usual classroom curriculum or other activities aimed at health education or literacy on alcohol, thus evaluating the effectiveness and cost-effectiveness of chatbots in promoting alcohol-related health literacy.

The development of smart conversational agents is done in multiple stages. At each new version, additional features and improvements can be added based on the performance of the previous version. Pahola was updated in 2022 to include more evidence-based information, additional branches, curated conversations, recall of the session to enable summary feedback to the user, an expansion of the quiz branch to include a complete brief intervention (the first version had only a brief advice), and new features to increase engagement and empathy. The new version was released in French as well.

### Limitations

Despite the promising scenario regarding using conversational agents to foster alcohol health literacy, there are multiple limitations linked to Pahola. The NLP engine is not perfect and depends on a minimum internet speed connection to run smoothly. Variations in internet speed can contribute to a decrease in the user experience leading to frustration. A better understanding of the user experience is still pending. Currently, efforts are being made to improve the solution’s performance and develop qualitative studies to better characterize the user experience.

### Conclusions

Traditional ways of improving health literacy related to school curricula and mass media campaigns were already known to be very limited, especially because they happen in environments with limited alcohol policy and must compete with the massive marketing of alcohol, which is often associated with success, health benefits, socialization, and culture. Our preliminary findings highlighted a promising scenario from using artificial intelligence solutions to tackle health education regarding alcohol harms. The most important goal of promoting higher levels of alcohol health literacy is to increase people’s knowledge and ability to make informed decisions regarding their alcohol consumption but also to support policies that will make their decisions easier, in an environment that has less pressure to drink. The initial data concerning Pahola’s reach have demonstrated its potential but more research and investment will be needed to turn it into a global public good.

## Abbreviations

NLP: natural language processing
PAHO: Pan American Health Organization
WHO: World Health Organization
